# Children's role in the community response to HIV in Zimbabwe

**DOI:** 10.7448/IAS.16.1.18468

**Published:** 2013-02-04

**Authors:** Morten Skovdal, Sitholubuhle Magutshwa-Zitha, Catherine Campbell, Constance Nyamukapa, Simon Gregson

**Affiliations:** 1 Department of Health Promotion and Development, University of Bergen, Bergen, Norway; 2 Biomedical Research and Training Institute, Harare, Zimbabwe; 3 Institute of Social Psychology, The London School of Economics and Political Science, London, UK; 4 Department of Infectious Disease Epidemiology, Imperial College London, London, UK

**Keywords:** childhood, agency, critical enablers, HIV, Investment Framework, Africa

## Abstract

**Introduction:**

Recent debates on how to achieve an optimal HIV response are dominated by intervention strategies that fail to recognize children's role in the community response to HIV. Whilst formal responses are key to the HIV response, they must recognize and build on indigenous community resources. This study examines adult's perspectives on the role of children in the HIV response in the Matobo District of southern Zimbabwe.

**Methods:**

Through a mix of individual interviews (*n*=19) and focus group discussions (*n*=9), 90 community members who were active in social groups spoke about their community response to HIV. Transcripts were subjected to a thematic analysis and coding to generate key concepts and representations.

**Findings:**

In the wake of the HIV epidemic, traditional views of children's social value as domestic “helpers” have evolved into them being regarded as capable and competent actors in the care and support of people living with HIV or AIDS, and as integral to household survival. Yet concurrent representations of children with excessive caregiving responsibilities as potentially vulnerable and at risk suggest that there is a limit to the role of children in the HIV response.

**Conclusion:**

Community volunteers and health staff delivering HIV services need to recognize the “behind the scene” role of children in the HIV response and ensure that children are incorporated into their *modus operandi* – both as social actors and as individuals in need of support.

## Introduction

In June 2011, coinciding with the United Nations high-level meeting on HIV/AIDS, a policy study published in *The Lancet* presents an Investment Framework for a more effective HIV response [1]. The Framework argues for a more focused and strategic use of scarce resources and calls for more cost-effective investment in basic HIV programme activities, which include the facilitation of “critical enablers”, defined as programmatic responses that improve the HIV response, and the promotion of synergies between the HIV and development sectors [1, 2]. The Framework recognizes the importance of the community response to HIV and promotes community mobilization and community-driven engagement activities (e.g. stigma reduction programmes and construction of health-enabling masculinities) as complementary strategies to basic HIV programming. Whilst this is important, the point of the departure of the Investment Framework is that “critical enablers” for a more effective HIV response, even when rooted in social processes (e.g. community mobilization), require some form of formal response (external intervention). This focus on formal responses has the potential to overlook indigenous community responses to HIV – from which much can be learnt to contextualize interventions and make them appropriate for local settings – that play a critical and enabling role in the HIV response [3]. Furthermore, reflecting the fact that the majority of community-based programmes are initiated and run by adults, and not children, the literature describing the Framework fails to recognize the role of children in the HIV response. We therefore ask the questions: What is the role of children in the HIV response? How do children fit into the Investment Framework?

Communities facilitate the HIV response both innately, through the everyday role of families and community members in supporting each other through life's challenges [4] and through participation in the implementation of programmes initiated by more resourceful organisations (e.g. health services, NGOs and CBOs) [5, 6]. A classic example of how resourceful organisations can enable community members to respond to the needs of those affected by HIV is through community health or adherence support workers [7, 8]. The HIV epidemic has given rise to an army of community volunteers who, at the interface between the health system and local realities, perform a critical role as social enablers in the HIV response [9]. With only a few exceptions [e.g. 10, 11], most HIV interventions drawing on community volunteers rely on adult community members. As such, adults get the most recognition as facilitators of HIV and AIDS care. Yet, a growing body of evidence suggests that, away from the limelight, children play a key role in the care and support of AIDS-sick adults and elderly grandparents [12–18]. Drawing on interviews with caregiving children, this work has highlighted who they care for (e.g. siblings, parents, grandparents), what caregiving roles they perform (e.g. nursing duties, income generation and domestic duties) [16–18], how they cope with hardship [12, 14], as well as the impact of caregiving on their school attendance [19] and psychological health [13, 20, 21]. This literature has predominantly brought forward children's perspectives. Incorporating children's voices into the debate is both a moral obligation and a pragmatic strategy for an improved HIV response. However, equally, we must not under-estimate the centrality of local adult's recognition of children's role in the HIV response. As adults continue to dominate the community response to HIV, their understandings of the role of children in the HIV response play a deciding role in availing opportunities that ensure children are integrated into wider local efforts – both as contributors to the HIV response and as individuals in need of support.

Therefore, there is a need to study and understand adult's representations of children's social value in the context of HIV. Traditionally, studies into the social value of children have sought to explain why some parts of the world have higher rates of fertility than others, highlighting the importance of the economic (and utilitarian), sociocultural and emotional value of children [22–24]. The nature of children's social value is intrinsically linked with household needs. Zelizer [25, p. 96], for example, traces how children evolved from their 19th century role as contributors to the household economy, to their 20th century status as economically “worthless”, but emotionally “priceless”. In poor rural communities of sub-Saharan Africa, where social welfare services are either non-existent or scarce, children continue to have a high economic value through their contribution to household livelihoods and the care of family members [15, 26–28].

To map out the social value of children in a high HIV prevalence and poor resource context, we draw on social representations theory (SRT) [29] to frame this study. Moscovici [30, p. xiii] defines social representations as “systems of values, ideas and practices with a two-fold function: first to establish an order which will enable individuals to orientate themselves in their material and social world and to master it; and secondly to enable communication to take place among the members of a community by providing them with a code for social exchange and for naming and classifying the various aspects of their world and their individual and group history”. In other words, SRT is concerned with the culturally-shared values, attitudes and practices that make up the representational field, or systems of socially constructed common sense knowledge, from which people learn to navigate through their social world. Social representations are properties of social groups, and not individuals, making this a particularly useful theory for the study and mapping of dominant representations pertaining to the social value of children in a particular context.

It is against this background, and in our interest to understand what the Investment Framework can do for children in the HIV response, that this study reports on interviews with adults and examines their social recognition and acceptability of children as actors in the HIV response.

## Methodology

This study draws on findings from a qualitative study that forms part of an on-going research project with ethical approval from the Medical Research Council of Zimbabwe (A/681) and Imperial College London (ICREC_9_3_13). The purpose of the current study was to explore community responses to HIV in Matabeleland South. Informed and written consent were gathered from all research participants on the condition that their identities would not be revealed. Pseudonyms are therefore used throughout.

### Study area and participants

The study took place in the Matobo District of Zimbabwe. The District has a population of 110,000 people and an HIV prevalence rate amongst women attending antenatal care at District clinics of 20%. The District has registered 3,623 people living with HIV or AIDS as receiving ART and recorded a total of 9,600 orphaned children – most of whom have been orphaned by AIDS-related illnesses.

Matobo is located in the Matabeleland South Province. The northern part of the District is characterized by an arid landscape, making cattle and goat keeping the primary source of income for residents. The south of the District offers greater opportunities for small-scale and subsistence farming. The District borders South Africa to the south and Botswana to the west, whose industry, cash crop farming and mining companies attract a significant number of Matobo men to look for work. While this sometimes results in the transfer of much needed funds to Matobo District, the migration of spouses presents serious challenges to HIV prevention, mitigation, treatment and care services, with some men discontinuing their treatment, and children taking on a greater role in sustaining their households.

We recruited 90 community group members to participate in this study. Participants were identified by researchers from the Biomedical Research and Training Institute in consultation with community guides and a representative from the District AIDS Action Committee. The study participants were over 18 years of age and members of a church group, AIDS support group, burial society, rotating credit society, a women's group, sports club, youth group, co-operative and a farmer's group (see Table 1).

**Table 1:** Community groups in Manicaland, eastern Zimbabwe

Group	Description
Church group	Members from the same congregation meet outside of regular church worship times. Engage in Bible study, discussing marital issues, and community outreach, particularly helping families in need (such as those with sick members or orphaned children).
AIDS support group	Loose term to apply to variety of groups, including post-HIV test clubs (mostly PLWHA), HIV/ART support groups often organized by clinics, youth groups, peer education groups, home-based care groups (members go house to house helping families with sick relatives – doing chores, bathing the sick, sometimes collecting pills from clinic, etc.).
Burial society	Members contribute small sums of money to central fund to cover basic funeral expenses of themselves and other members. Members commit to organizing proper burials for one another and often sing at funerals. Generally meet monthly.
Rotating credit society	Members contribute to central fund and when they reach a certain amount the money is shared for income generating projects such as buying seeds. Members borrow at same interest rate and loans can be made to non-members at a higher rate.
Women's group	Often linked to government women's empowerment initiatives. Supported by government income generating grants.
Sports club	Male dominated. Organize tournaments against other regions. Primarily soccer.
Youth group	Often organized by political parties or teachers, these seek to develop leadership skills and provide recreation for youth (often into 20s – “end of youth” often determined by marriage).
Co-operative	Group members come together to set up an income generating project, co-owned and run by members. The groups sometimes get assistance from NGOs to expand their work.
Farmer's group	Farmers, both male and female, meet monthly to plant crops, discuss weather patterns and new technologies, share labour and access NGO assistance (e.g. for farming implements or water irrigation).

### Data collection and analysis

From each of the nine different social groups participating in this study, we interviewed group members through in-depth interviews (IDIs) and focus group discussions (FGDs) (see Table 2). Interviews were conducted in the group's regular meeting area by trained and experienced researchers who carried out the interviews in the local Ndebele language. Interviews were digitally recorded.

**Table 2:** Participant characteristics

Type of informants	IDIs	FGDs	Total
AIDS support group members	2 women, 1 man	1 (8 women and 1 man)	12
Burial society group members	2 women	1 (4 women and 2 men)	8
Church group members	1 woman, 1 man	1 (11 women)	13
Cooperative members	1 woman	1 (7 women and 1 man)	9
Farmers group members	2 women, 1 man	1 (4 women and 5 men)	12
Savings and lending group members	3 women	1 (5 women and 1 man)	9
Soccer club members	3 men	1 (8 men)	11
Women's group members	2 women	1 (4 women)	6
Youth group members		1 (5 women and 5 men)	10
Total no. of participants	19	9 groups (71 participants)	90

Individual interviews and focus group discussions lasted approximately 90 and 120 minutes, respectively. To compensate for their time, we provided each participant with two bars of soap, lunch and reimbursement of transport costs.

A single topic guide was used to frame both interview methods. It was designed to explore the role of community membership and dialogue in encouraging engagement with HIV prevention, mitigation and care efforts, as well as community strategies, strengths and resources available to support people affected by HIV. Only one out of the 38 open-ended questions listed on the topic was directed at the role of children in the HIV response: “What is the role of children in contributing to the survival of households affected by HIV?” Despite there only being one question on the topic guide pertaining to the role of children, this was a topic that sparked considerable discussion across all of the groups, particularly in the focus group discussions where participants continued to build on each other's response to the question.

Audio recordings were transcribed and translated from Ndebele to English and imported into Atlas. Ti, a software package designed for qualitative data analysis. Transcripts were read carefully before the coding process started. A total of 96 codes, encompassing 907 text segments, or quotations, emerged from this process – detailing community responses to HIV. This paper does not seek to report on the entire data set, but instead explores the surprisingly prominent response to the question on children's contributing role in the HIV response. As a result of the lengthy responses and discussions arising from that single question, 15 codes, encompassing 74 text segments (8% of all data), give detail to adult's perspectives of children's role in the HIV response. Following Attride-Stirling's [31] thematic network analysis, codes were progressively and analytically grouped together into more interpretative global themes (see Table 3). Reflecting our social representations approach, the themes generated reflect dominant views as opposed to individualized personal experiences or differences between sub-groups (e.g. gender or HIV status of respondents). We will now discuss each of the basic themes emerging from our data under headings reflecting our global themes.

**Table 3:** Thematic network: Contextualizing children's response to HIV

Global themes	Organizing themes	Basic theme (codes)	Illustrative text segments from transcripts
Children as a community resource	Children and domestic duties	1. Children serve a role as domestic helpers, sustain livelihoods through food and income generation	“… I have a child. He is the one who goes to the fields and helps other people and he would come back with food …”
		2. Children help care for siblings	“… they will take care of each other, like that the older ones will be taking care of the little ones …”
	Children as social protection for old age	3. Children serve as life insurance	“… If I do not have children I will not live …”
		4. Children are better able to do jobs requiring strength and can thus help elderly guardians with daily living	“… most of the gardening is being carried out by my grandchildren …”
		5. Caregiving is an act of reciprocity	“… If you have raised them [children] well they will remember you and they will also help you …”
In response to the HIV epidemic, children take on significant caregiving roles	Children caring for parents living with HIV or AIDS	6. Children sustain and care for HIV sick parents	“… I was bedridden […] my children sustained me …”
		7. Children provide emotional support for HIV sick parents	“… they [children] have the strength to give us ‘emotional’ support. They will say soothing words of comfort to the patient …”
		8. Children seek social support to sustain HIV-positive households	“… children will tell the neighbours that today my mother is ill in order to get help …”
	Children help with ART adherence	9. Children remind HIV-positive parents to take their drugs	“… children even remind us when we forget to take our drugs …”
		10. Children fetch antiretroviral drugs	“… children can go and collect medication …”
		11. Children work with treatment partners	“… If I am getting late, even by just a minute, they [children] will come and fetch me …”
Community members recognize the limitations of young caregiving	Children should not be caring	12. Younger caregivers are vulnerable and should not care	“… caregiving becomes a burden for the children and makes them very vulnerable …”
		13. Not all adults recognize children as social enablers	“… If the father and mother are ill, then there will be hunger in the home …”
	Children who are not looked after engage in “bad” behaviour	14. Adult supervision and control is important	“… If the parents are ill […] the children will just do as they want …”
		15. Poor and orphaned children engage in risky behaviours	“… they may end up stealing […] they may sell their bodies so that they get food …”

## Findings

### Children as a community resource

Children are an important community resource. In Matabeleland, adult community members often spoke of children as “helpers”, whether it was their biological children or those orphaned and in foster care. A number of examples of how children contribute to their household livelihood were provided. For example, children, particularly those a little older, were said to help out with child care (e.g. of younger siblings), domestic duties as well as food and income generation for the household.

The way they can help is that they will take care of each other, like that the older ones will be taking care of the little ones, they can clean the house and take care of the home, they can take the little ones to school that is how they can help. Bheka (male, age 62, member of a burial society, FGD)

I do have children. One of them is the young man. He is the one who goes to the fields and helps other people and he would come back with food. Gugu (female, age 62, member of an HIV/AIDS support group, IDI)

Although older children were seen as more productive to the household, younger children, as part of their socialization and training, were also said to help out with domestic duties, such as cleaning, washing of clothes and cooking.

The role of children as helpers to their family unit follows them throughout their life. *Ukuzala yikuzimbela* is a local Ndebele saying which means that “having children is like storing for the future and of benefit to oneself”. It captures the essence of how many adults in poor and rural communities of Matabeleland describe the importance of children. Children are seen as an insurance policy, and play a critical role, particularly in the context of limited social welfare for elderly people, in the care and support of fragile parents or grandparents.

Children are your future. If I do not have children I will not live. If I fall ill, a child will nurse me, if I get a stroke and I cannot stand up, or become blind, they will help me. Owethu (female, age 65, member of a burial society, FGD)

Having children is a very wonderful thing. As an elderly person, you will find that most of the gardening we do is being carried out by my grandchildren while I attend to other issues. Secondly, as the days go by and my age finally catches up with me, I will be able to retire and enjoy the fruits of my labour while my grandchildren take care of me. That is the wonderful thing about having children. Sindani (male, age 69, member of a farmers’ association, FGP)

For some people, like Owethu and Sindani, children can have a lifesaving role. This is a potentially important motivation for some elderly people to agree to foster orphaned children. Bheka talks about caregiving as an act of reciprocity, suggesting that orphaned children who are raised well by their foster parents are likely to reciprocate the care and support they have received when illness or old age prevents their foster parents from taking care of themselves.

If you have taught them well, they know good from bad. You may care for a child that is not biologically yours. If you have raised them well they will remember you and they will also help you. If you have reached a stage where you are old, blind and you can no longer do anything by yourself, they will be doing it for you, quickly. Bheka (male, age 62, member of a burial society, FGD)

Families in Matabeleland South have struggled with drought and poverty for centuries, making children an integral part of household survival. This makes children valuable, particularly for households with adults increasingly less able to perform basic household tasks. We now explore how children's role as “helpers”, qualifies them as critical enables in the HIV response.

### Children's caregiving of adults living with HIV

A number of examples of children's caregiving emerged from the interviews. Children were praised for providing significant nursing care and support for their sick and bedridden parents. Despite their young age, they were seen as competent carers by many of the respondents.

There are children who nurse adults. Not every household has an adult caregiver and then you will find that the sick parent is being nursed by a child. You will find that, despite the age, the child is able to nurse the parent back to health. They are a great help, we cannot find fault in them. Sibusisiwe (female, age 27, member of a savings and lending club, FGD)

HIV-positive respondents gave examples of how their own children had taken on the responsibility of sustaining them through illness, for example, by keeping them clean, feeding them and keeping the household running. Sitheni, a 53-year-old woman, explained how her children provided her with life-saving support during times of illness.

Having a child is very good. I was bedridden and spent four months unable to do anything. My children sustained me. Even today you find the place clean. It is the boys who have cleaned. I wake up and they make sure I eat. If I did not have these children I do not know what state I would be in. I am alive because of these children and for that I am thankful. Sitheni (female, age 53, member of a women's group, FGD)

Children were also said to work directly with the health services to ensure their parents adhere to antiretroviral therapy. Numerous examples were given of how children reminded their parents to take the drugs, eat and rest well. Children were reported to fetch antiretroviral drugs from clinics, giving them an opportunity to report to nurses how their parents are doing and to pass on general advice – taking on the role as a treatment partner.

People who are ill and who have children can be assisted by children. They can go and collect medication and remind their parents to take the medication, as well as giving them general advice. Mehluli (male, age 24, member of a youth group, FGD)

A couple of community health workers, or treatment partners, spoke about how children work with them to ensure their parents adhere to their treatment.

Children are helpful. I have a neighbor who is ill. She stays with 2 children, a boy and a girl, but when you go to their home you will find that the children are taking very good care of their mother. I visit her to mark the register for her ART pills. If I am getting late, even by just a minute, they will come and fetch me and I will run to their home. Sihle (female, age 47, member of a women's group, FGD)

Children were not only recognized as providers of nursing care and support, they were also said to have the strength and maturity to provide sick adults with emotional support, listening to their worries and providing them with encouragement.

What I have observed is that although children are not able to do much, they have the strength to give us ‘emotional’ and ‘physical’ support. They will fetch water or say soothing words of comfort to the patient. They are able to lend an ear and listen, although they cannot afford to take you to the hospital because they do not have money, they are able, in their own way, to provide great support. Sithabisile (female, age 28, member of a farmers’ association, FGD)

The quote by Sithabisile illustrates how many adults in this community recognize children as important actors in the support of people living with HIV and AIDS. In cases where they may juggle with school commitments and caregiving, they were often able to negotiate social support, such as from neighbours.

Children are really supportive. They are the ones who will be fetching water, looking for firewood and tell the neighbours that today my mother is ill in order to get help. Senzokuhle (female, age 56, member of an HIV/AIDS support group, FGD)

In addition to caring for their parents, orphaned children were reported to care for their sick foster parents. HIV-positive adults abandoned by their own children may find it beneficial to foster orphaned children, knowing they will take care of them in return for fosterage.

Fostering children is good because they are helpful. Your own children may grow up and leave you and then the ones who are not yours will stay and take care of you, they help a lot at home. Dalitso (female, age 45, member of HIV/AIDS support group, FGD)

Children are committed to keeping their parents, or foster parents, alive and healthy and actively engage in activities that sustain their health. Most of the adults interviewed for this study recognized the positive role children play in caring for adults living with HIV and AIDS. However, in addition to recognizing the important role of children in the HIV response, many acknowledged the limitations of seeing children as critical enablers in the HIV response.

### Limitations of young caregiving

A number of respondents felt that children should not be engaging in the caregiving of AIDS-sick parents, arguing they had little choice in the matter and faced significant struggles, in ways that potentially undermined their well-being.

In families where parents are ill, children face difficulties on how to take care of their parents. It becomes a burden for the children and makes them very vulnerable. Thando (female, age 51, member of a burial society, FGD)

Similarly, Amahle argues that a child with two sick parents will suffer because there is nobody to generate income or food.

If the father and mother are ill, then there will be hunger in the home because the parents are the ones who have to look for food and the children will not go to school. Amahle (male, age 23, member of a HIV/AIDS support group, FGD)

Other respondents felt that children living in households where the adults were bedridden and thus unable to supervise them were at heightened risk of engaging in “bad” behavior. Numerous examples were given of how children living in HIV-positive households with labour-constrained adults were more likely to steal and prostitute themselves for survival.

If the parents are ill, then there is nothing good there because even the children have no one to control them or reprimanding them, how can you do that when you are bedridden, so the children will just do as they want. Mehluli (male, age 24, member of a youth group, FGD)

Child-headed households, or those with a single and sick parent are likely to go hungry, have no clothes or money for fees. They may end up doing things that will put them at risk of getting HIV. They may end up stealing. If it is a small child and they have no source of income, they may sell their bodies so that they get food even though they have already lost their parents to HIV. Senzokuhle (female, age 56, member of an HIV/AIDS support group, FGD)

## Discussion

In light of growing international recognition of the vital contribution of the community response to HIV management and impact mitigation, we explored community members’ views of the role of children in responding to HIV. Our findings suggest that children in this rural and poor context, as in many other parts of sub-Saharan Africa [15, 32–34], are recognized as an important resource in sustaining household livelihoods, with children helping out with domestic duties and contributing to caregiving, food and income generation. Children were seen as “helpers” and considered a safety net during illness and old age, a recognition that has also been identified by children in Kenya [12, 15]. In the wake of the epidemic, traditional views of children as domestic “helpers” have evolved into them being regarded as capable and competent actors in the care and support of people living with HIV or AIDS, and as integral to household survival. Children were reported to take on significant caregiving responsibilities, feeding and washing their bedridden parents as well as providing them with emotional support. Children were said to take on the role of treatment partners for their parents, fetching drugs from local health clinics and ensuring timely adherence, resonating with reports from children in Zimbabwe [35] and in Kenya [14, 18]. The supportive role of children has also been observed in the United States, where HIV-positive women with children have reported better psychological functioning compared to their childless peers [36].

Although our respondents recognized the valuable role of children and articulated their deep respect for them, many also raised a concern about the situation many children in HIV-positive households frequently find themselves in. They acknowledged that children often had excessive caregiving duties and did not have much of a choice in whether or not they want to take on a role in the HIV response, as this is dictated by their living arrangements and family circumstances. Children in AIDS-affected households were often perceived to be without adequate adult guardianship and therefore more at risk.

This study highlights a certain ambivalence concerning children's role in the HIV response. On the one hand, children living with HIV-positive adults were said to save lives and were described as competent, faultless, responsible, caring and strong children. On the other hand, the realization that many children in AIDS-affected households are vulnerable indicates that there is a limit to community members’ views of the role that children should play in the HIV response. This unresolved ambiguity underpinned our informants’ views of the social acceptability of children's role in the HIV response. While it may be acceptable for a child to be socialized as responsible and helpful in the HIV response, this does not automatically make it acceptable for children to be the sole caregivers and run a household without adult support and supervision. Furthermore, just because a child exhibits ingenuity and skill in the caregiving of adults, it does not mean that this is the most favourable situation for the child. Nonetheless, what these findings demonstrate is that, irrespective of adults’ views of the acceptability of this situation, in these low-resource and high prevalence communities of Zimbabwe, children *do* play a critical role in the HIV response.

### So what does this mean for the Investment Framework?

Our findings highlight the urgent need for better international recognition of the role of children as actors in the HIV response. Efforts to understand and strengthen the role of communities in facilitating more effective uptake and use of HIV services, through understanding and facilitating community contributions to activities such as home-based care and adherence support, need to acknowledge the *de facto* role that children are playing as “critical enablers”. However, important as it is to see children as social actors in the HIV response and chart out the different ways in which children contribute to the HIV response, this should not be used as justification to seeing them as a free resource that can be tapped on by the health system. As suggested by our informants, many children living in a household affected by HIV and AIDS are vulnerable and in need of support. As such, this recognition of children as “critical enablers” should be seen as an opportunity to ensure that HIV programmes (e.g. home based care, adherence support, livelihood support) strengthen families and support all household members, rather than narrowly focusing on the HIV-positive person – a recommendation also made by the Joint Learning Initiative on Children and AIDS [37]. Community volunteers and health staff delivering HIV services need to recognize the “behind the scene” role of children in the HIV response and ensure that children are supported in their caregiving role (e.g. through home based care training, provision of gloves, psychosocial support, provision of household assets or cash transfers) both as a means to address their needs as caregivers and to respect them as partners in the HIV response.

## Conclusion

We conclude that there is an urgent need to incorporate children of HIV-positive adults into basic programme designs and encourage a synergy between HIV programmes and social protection strategies targeting children affected by HIV. To move this agenda forward in a way that resonates with local responses to the needs of children of HIV-positive parents, future research needs to develop in-depth understandings of indigenous support structures for children living in low resources and high HIV prevalence communities and use this as a platform to bolster those support structures and the effectiveness of HIV programmes.
